# Genetic Architecture Underlying Response to the Fungal Pathogen *Dothistroma septosporum* in Lodgepole Pine, Jack Pine, and Their Hybrids

**DOI:** 10.1111/eva.70078

**Published:** 2025-02-06

**Authors:** Mengmeng Lu, Nicolas Feau, Brandon Lind, Dragana Obreht Vidakovic, Pooja Singh, Sally N. Aitken, Richard C. Hamelin, Sam Yeaman

**Affiliations:** ^1^ Department of Biological Sciences University of Calgary Calgary Alberta Canada; ^2^ Department of Biological Sciences University of Notre Dame Notre Dame Indiana USA; ^3^ Department of Forest and Conservation Sciences University of British Columbia Vancouver British Columbia Canada; ^4^ Canadian Forest Service, Pacific Forestry Centre, Natural Resources Canada Victoria British Columbia Canada; ^5^ Aquatic Ecology & Evolution Division, Institute of Ecology and Evolution University of Bern Bern Switzerland; ^6^ Center for Ecology, Evolution & Biogeochemistry, Swiss Federal Institute of Aquatic Science and Technology (EAWAG) Kastanienbaum Switzerland; ^7^ Institut de Biologie Intégrative et Des Systèmes Université Laval, Pavillon Charles‐Eugène‐Marchand 1030 Québec City Québec Canada

**Keywords:** conifer, *F*_ST_, fungal pathogen resistance, hybrid, introgression, pool‐GWAS

## Abstract

In recent decades, *Dothistroma* needle blight (DNB), a pine tree disease caused by the fungal pathogen *Dothistroma septosporum,* has severely damaged lodgepole pine ( *Pinus contorta* Dougl. ex. Loud.) in British Columbia, Canada, and raised health concerns for jack pine ( *Pinus banksiana* Lamb.). The pathogen has already shown signs of host shift eastward to the hybrid populations between lodgepole pine and jack pine ( *Pinus contorta* × *P. banksiana* ), and possibly into pure jack pine. However, we have little knowledge about mechanisms of resistance to *D*. *septosporum*, especially the underlying genetic basis of variation in pines. In this study, we conducted controlled inoculations to induce infection by *D. septosporum* and performed a genome‐wide case–control association study with pooled sequencing (pool‐seq) data to dissect the genetic architecture underlying response in lodgepole pine, jack pine, and their hybrids. We identified candidate genes associated with *D. septosporum* response in lodgepole pine and in hybrid samples. We also assessed genetic structure in hybrid populations and inferred how introgression may affect the distribution of genetic variation involved in *D. septosporum* response in the studied samples. These results can be used to develop genomic tools to evaluate DNB risk, guide forest management strategies, and potentially select for resistant genotypes.

## 1 Introduction

Pests and pathogens constantly threaten forest trees, and their impacts are changing as climates are altered (Seidl et al. 2017; Simler‐Williamson, Rizzo, and Cobb 2019). Knowledge about the genetic architecture of pathogen resistance and the identified candidate resistance (R) genes provides the basis for genomic breeding to improve tree resilience to forest pathogens. Genes and alleles that confer disease resistance or tolerance can eventually be used to develop genomic tools for rapidly selecting resistant genotypes (Isabel, Holliday, and Aitken 2019). Current breeding practices for forest trees involve recurrent selection, which includes repeated cycles of selection, breeding, and testing to search for genotypes with better disease resistance, and then deployment of these selected tree genotypes (White 2004). This takes years of effort to design and implement. Increasingly, genome editing is used to develop crops resistant to disease (van Esse, Reuber, and van der Does 2020). For fruit trees, the CRISPR/Cas9 system has been applied to enhance disease resistance in citrus (Jia et al. 2017; Wang et al. 2019), apples (Zhou et al. 2020), and others (Min et al. 2022). Given the long generation time in conifers and the frequent threats from diseases and insects, identifying R genes that can be targeted for selection or editing is a useful step in developing resistant genotypes for reforestation.

*Dothistroma* needle blight (DNB), which is caused by the fungal pathogen *Dothistroma septosporum*, is a foliar disease of a wide range of pine trees (Gibson 1972), with infected trees showing reddish‐brown bands in needles, defoliation, and growth reduction. DNB has caused increased damage to lodgepole pine ( *Pinus contorta* Dougl. ex. Loud., LP) in recent decades in Western Canada (Dale, Lewis, and Murray 2011; Woods 2003) and is now expanding its range eastwards, threatening jack pine ( *Pinus banksiana* Lamb., JP). In severe cases, DNB causes extensive mortality, even amongst mature trees in LP plantations (Woods 2003). Historically, DNB had only minor impacts on native forest trees in North America, but emerged as a severe forest disease during the 1950s to 1960s in *Pinus radiata* plantations in Africa, New Zealand, and South America (Gibson 1972). Recently, DNB has caused increased outbreak incidence and host range expansion in North America and Europe, which is likely correlated with climate change (Boroń et al. 2021; Welsh, Lewis, and Woods 2009; Woods, Coates, and Hamann 2005). In North America, while *D. septosporum* tends to be more common in moist western forests closer to the Pacific Ocean, *Dothistroma*‐like symptoms were observed in drier forests of LP and natural hybrids between LP and JP ( *Pinus contorta* × *P. banksiana*, LP × JP) in northern Alberta in 2012 and 2013 with further tests confirming *D. septosporum* was the cause of the disease observed (Ramsfield, Myrholm, and Tomm 2021). Feau et al. (2021) performed a controlled inoculation experiment and demonstrated that LP, JP, and LP × JP are susceptible to *D. septosporum*, with JP showing a higher disease severity than LP or LP × JP, though there is no report showing DNB infection in natural JP forests. Considering *D. septosporum*'s eastward shift reaching towards the natural range of JP and JP's high susceptibility to *D*. *septosporum*, it is important to understand how these species respond to DNB infection.

The evolutionary history and biogeography of JP and LP have likely played an important role in shaping the genetic basis of resistance to DNB. JP is widely distributed across boreal forests in eastern North America, extending in Canada from Northwest Territories to Nova Scotia (Rudolph and Laidly 1990), areas largely outside of the historical range of *Dothistroma*. LP and JP evolved in allopatry, with an estimated divergence time of ~8 million years before present (Hao et al. 2015), but hybridized in areas of contact, primarily in central and northwestern Alberta (Burns et al. 2019; Rudolph and Yeatman 1982). Following secondary contact of these two species, additional patchy regions of introgression have been developed in central Alberta north through to the Northwest Territories and northeastern British Columbia to the Alberta‐Saskatchewan border (Burns et al. 2019). Natural LP × JP hybrids display a wide range of phenotypes that are intermediate or more typical of one parent, depending on the level of introgression (Wood, Hartley, and Watson 2009; Yeatman and Teich 1969).

In light of the geographic distribution of LP and JP, the longer history of host‐pathogen coevolution in LP might explain its stronger resistance to DNB infection compared to JP. A former study has evidenced coevolution between *Cronartium harknessii* lineages with LP and JP, with genetic basis underlying both pathogen virulence and host resistance (McAllister et al. 2022). For DNB infection caused by *D*. *septosporum*, though we lack direct evidence to prove a coevolutionary history between *D*. *septosporum* and different pine hosts, we have already identified candidate resistance genes to *D*. *septosporum* by RNA‐seq analysis on experimentally infected LP seedlings (Lu et al. 2021). These candidate genes include 43 genes that are present in the plant‐pathogen interaction pathway, as well as nine R genes that contained sites under positive selection. It seems that both constitutive (baseline) and induced (defenses activated after being attacked) defenses have been developed in LP. However, little is known about the genetic basis of resistance to *D*. *septosporum* in JP. When subjected to other biotic agents, LP and JP showed unique monoterpene profiles in response to mountain pine beetle (Hall et al. 2013) and significant differences in chitinase gene expression in response to the fungal pathogens *Grosmannia clavigera* and *Cronartium harknessii* (Peery et al. 2021). These previous studies revealed the species‐specific pathogen responses in a focused set of gene families. Nevertheless, since many complex traits are determined by many genes of small effect, to identify the causal genes underlying disease response traits, a genome‐wide gene survey might give us a high‐resolution perspective (Alonso‐Blanco and Méndez‐Vigo 2014; Tam et al. 2019).

To dissect the genetic architecture of pathogen resistance and to identify candidate genes, researchers have employed genetic markers, mainly SNPs, and methods like quantitative trait locus (QTL) mapping and genome‐wide association analysis (GWAS). Polygenic disease responses, comprising numerous loci of small effect, appear to be the rule (Stocks et al. 2019). Large numbers of SNPs were associated with pitch canker resistance in *Pinus taeda* L. (De La Torre et al. 2019; Lu et al. 2017; Quesada et al. 2010), with white pine blister rust resistance in *Pinus lambertiana* Dougl. (Weiss et al. 2020), and with *Heterobasidion* root rot resistance in *Picea abies* (L.) Karst. (Capador‐Barreto et al. 2021). To identify the candidate R genes, most GWAS studies on forest trees employ individual‐based methods, with both phenotypic and genotypic data collected on each sampled individual. Case–control methods have been developed to study disease where individuals are pooled into healthy vs. infected groups, but such approaches typically still use individual‐based sequencing to call genotypes before pooling (Wu et al. 2010). When multiple replicates are available, it is possible to combine pool‐seq and the case–control design: Endler et al. (2016) analyzed the genetic differences underlying abdominal pigmentation variation among *Drosophila* populations, while Stocks et al. (2019) identified SNPs associated with low versus high ash dieback damage in *Fraxinus excelsior* L. using this approach. Recently, Singh et al. (2024) used a case–control pool‐seq approach similar to that deployed here to search for signatures of resistance to Swiss needle cast in *Pseudotsuga menziesii* (Mirb.) Franco. Pool‐seq is a cost‐effective alternative approach to sequencing of individuals, but the weakness and limitations are also evident, such as unequal representation of individuals and suboptimal allele frequency estimate when the pool size is small, as well as misaligned mapping and sequencing errors (Schlötterer et al. 2014). Nonetheless, in a study on conifer genotyping, Lind et al. (2022) found that allele frequencies estimated from pooled DNA sequencing samples were highly correlated with frequencies estimated from individual sequencing.

Another factor in understanding the genetics of resistance is whether there is interplay between any alleles conferring resistance and introgression between the species. Bechsgaard, Jorgensen, and Schierup (2017) showed that plant R genes can adaptively introgress between closely related species. The LP × JP hybrid zone has been very well studied by Cullingham et al. (2012), who found that this hybrid zone presents a mosaic zone with variable introgression and patchy distributions of hybrids (Burns et al. 2019). While it is clear that these species share a broad hybrid zone, it is unclear whether any alleles for disease resistance will be more or less introgressed than the average region in the genome.

In this study, our aim is to identify the genetic basis of DNB resistance in LP, JP, and LP × JP samples and to explore spatial patterning in any identified loci. The specific objectives of our study were to: (1) develop a GWAS case–control approach using pool‐seq samples to identify loci with consistent associations with *D. septosporum* response across replicates; and (2) compare the genetic architecture underlying DNB resistance within the studied pine trees and infer how introgression may affect the genetic basis of *D. septosporum* responses. This study provides an important step towards identifying candidate genes to develop genomic tools for screening trees resistant to DNB infection.

## 2 Methods

### 2.1 Plant Materials

**FIGURE 1:**
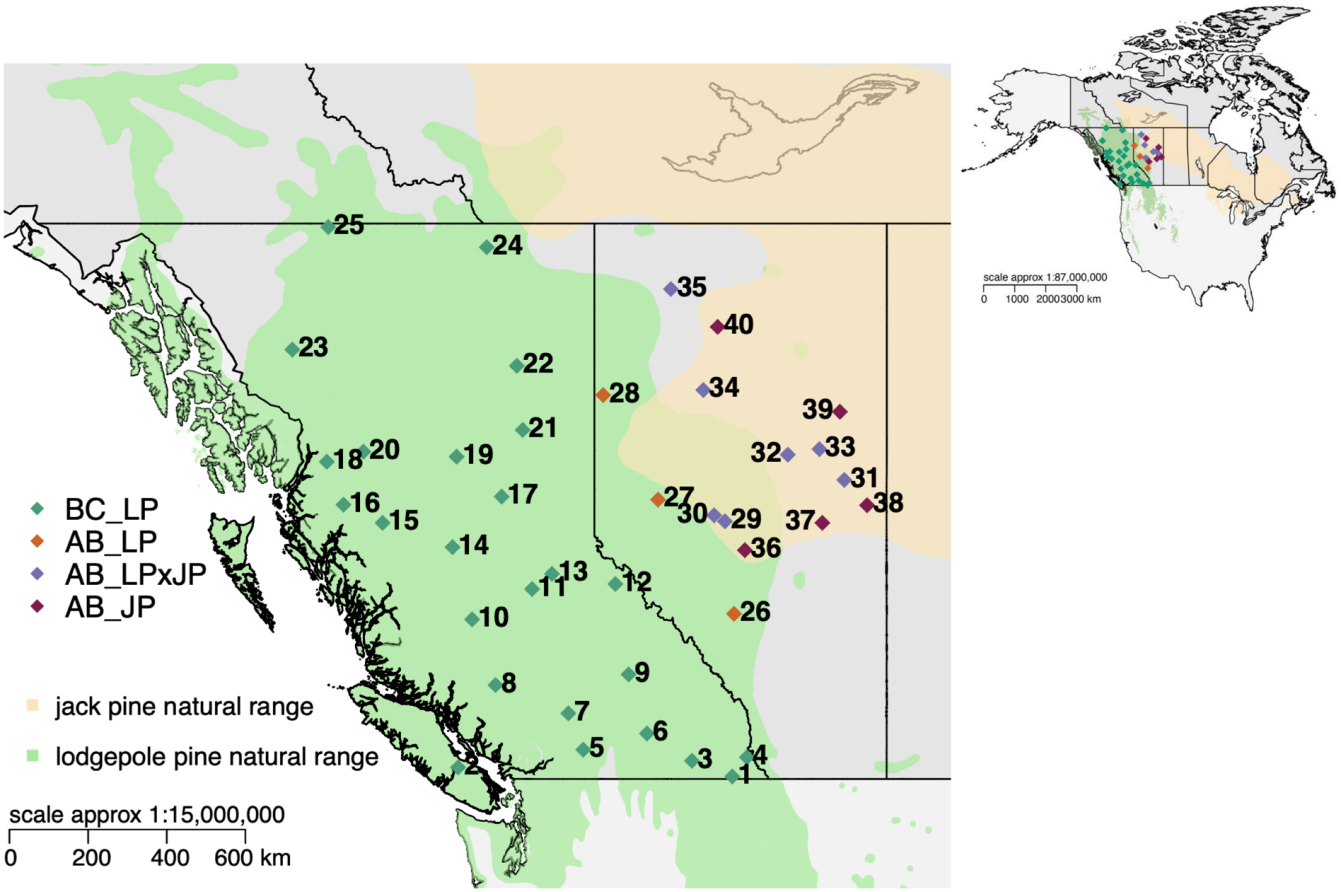
Sampling locations (Table S1) of the 40 pine seedlots analyzed in this study, plotted within the natural range of *Pinus contorta* (lodgepole pine) and *Pinus banksiana* (jack pine) in Canada and the USA. The top right map shows the context of the sampling locations. The range maps were downloaded from https://sites.ualberta.ca/~ahamann/data/rangemaps.html. BC_LP (green diamonds)— *Pinus contorta* seedlots from British Columbia (*N* = 25); AB_LP (orange diamonds)— *Pinus contorta* seedlots from Alberta (*N* = 3); AB_ LPxJP (purple diamonds)— *Pinus contorta* × *P. banksiana* hybrids seedlots from Alberta (*N* = 7); AB_JP (brown diamonds)— *Pinus banksiana* seedlots from Alberta (*N* = 5).

Seeds were obtained from 40 natural seedlots across Western Canada (Figure 1, seedlot numbers and locations can be found in Table S1, seed contributors http://adaptree.forestry.ubc.ca/seed‐contributors/), including 25 LP seedlots from British Columbia (BC_LP) and three from Alberta (AB_LP), seven LP × JP seedlots from Alberta (AB_LPxJP), and five JP seedlots from Alberta (AB_JP). The range maps were downloaded from https://sites.ualberta.ca/~ahamann/data/rangemaps.html (Hamann et al. 2005). When the seeds were collected in the wild, they were assigned to pure LP, pure JP, or LP × JP, based on their location and morphological traits such as cone and branch characteristics, microfibril angle, and cell area (Wheeler and Guries 1987; Wood, Hartley, and Watson 2009; Yeatman and Teich 1969). The proportion of LP and JP ancestry of the collected seeds were genotyped using 11 microsatellite loci by Cullingham et al. (2012). Briefly, seeds were germinated to obtain seedlings, then DNA was isolated from the seedlings. DNA was used to amplify 11 microsatellite loci and allele sizes were determined for genotyping as described by Cullingham et al. (2011).

For the present study, seeds from the 40 seedlots were used to grow seedlings in a greenhouse at University of British Columbia, Vancouver, BC, for their first year (a flow chart of experimental procedure is shown in Figure S1). Resistance was phenotyped by inoculating 100 individuals per seedlot with one of two *D. septosporum* isolates (D1 & D2, 50 individuals of each isolate). D1 was isolated from needles of an infected LP × JP seedlot in Alberta, Canada, while D2 was isolated from an infected LP seedlot in the Kispiox Valley region close to Smithers in Northwestern British Columbia, Canada (Feau et al. 2021; Ramsfield, Myrholm, and Tomm 2021). The seedlots were identified as LP or LP × JP as aforementioned (Cullingham et al. 2012). The controlled inoculation experiment was performed as described in Kabir, Ganley, and Bradshaw (2013) and Feau et al. (2021). Briefly, the one‐year‐old seedlings were placed in a completely randomized experimental design in a growth chamber with the condition of 16 h daylight at 20°C, 8 h a night at 12°C, and minimum relative humidity of 80%. *D. septosporum* conidia were harvested from colonies grown for 10–16 days on *Dothistroma* sporulation medium plates and then suspended in sterile distilled water. Each seedling was sprayed twice at 10‐day intervals with a standard inoculum of approximately 3 mL of 1.6 × 10^6^ conidia mL^−1^, using a household trigger action atomizer. Once the needles were dry (~45 min), the seedlings were wrapped in transparent plastic bags and kept in the growth chamber. After 48 h the plastic bags were removed and the seedlings were kept in the growth chamber for 15 weeks till phenotyping. A mister spraying tap water was activated hourly for 3 min during the trial to maintain needle wetness. Control seedlings, at least one for each seedlot, were inoculated with sterile water.

Fifteen weeks after inoculation, seedlings were rated for proportion of necrotic needles with red bands and/or fruiting bodies according to a disease severity scale of 1 to 5 (1 = least, 5 = most disease severity). Following disease rating, the 10 most‐ and 10 least‐infected individuals per seedlot (five individuals for each *D. septosporum* isolate) were identified and retained for DNA extraction. Genomic DNA of each of these individuals was extracted as described in Lind et al. (2022), using the Nucleospin 96 Plant II Core kit (Macherey–Nagel GmbH & Co. KG, Germany) on an Eppendorf epMotion 5075 liquid‐handling platform. The DNA of five most‐infected and five least‐infected individuals by each of the two *D. septosporum* isolates was combined in equimolar amounts to compose four pooled libraries per population, two susceptible (D1‐S, D2‐S) and two resistant libraries (D1‐R, D2‐R). Hence,160 pooled DNA libraries were generated.

### 2.2 Sequence Capture and Pool‐Seq Genotyping

The capture probes were comprised of two sets of probes. The first set of probes was designed based on an existing LP sequence capture array (Suren et al. 2016) by removing the probes that did not yield successful genotyping in Yeaman et al. (2016). Probe sequences of the existing capture array were aligned to the reference genome using GMAP v2019‐03‐15 (Wu and Watanabe 2005). Probes that covered the genomic regions with called SNPs in the dataset from Yeaman et al. (2016) were retained; otherwise, the probes were discarded. Since there is no available LP reference genome, a masked *Pinus taeda* reference genome, Pita.2_01.masked3k2.fa (https://treegenesdb.org/FTP/Genomes/Pita/v2.01/genome/; Neale et al. 2014), was used instead. *Pinus taeda* is a closely related species to LP (Jin et al. 2021). The second set of probes was newly designed probes derived from the *D. septosporum*‐induced genes, which were based on an LP reference transcriptome assembled using the RNA‐seq data of *D. septosporum‐*infected LP samples (Lu et al. 2021). To avoid duplicates, only those *D. septosporum‐*induced genes with low homology to the retained probe sequences were used to design the new probes. To do so, the retained probe sequences were aligned to this transcriptome using blastn v2.9.0 with an E‐value of 1e‐10. A total of 8778 *D. septosporum*‐induced genes did not have any aligned probe sequences. These non‐duplicate *D. septosporum‐*induced genes were subsequently aligned to the reference genome to predict the exon‐intron boundaries using GMAP v2019‐03‐15. Exon sequences from these induced genes with a length of at least 100 bp were combined with the previously designed working probe sequences, and this combined set of sequences was submitted to Roche NimbleGen (Roche Sequencing Solutions Inc., CA, USA) for custom SeqCap EZ probe design (design name: 180321_lodgepole_v2_EZ). Combining the two sets of probes, this updated LP sequence capture array has a capture space of 44 Mbp, containing roughly 35,467 assembled genes. Most LP genes responsive to environmental stress and fungal pathogen attack were included in the current capture probe design. Though genes expressed in different development periods might be missed, genes that have evidence of substantial expression have been covered in this capture probe design.

The capture libraries for each of the 160 pools were constructed following NimbleGen SeqCap EZ Library SR User's Guide and as described in Lind et al. (2022). Then the R (resistant) and S (susceptible) libraries (two libraries per capture, R1 + S1 or R2 + S2, indexed with different barcodes) per population and per isolate were combined for sequence capture and enrichment. Sequencing was performed using the Illumina NovaSeq 6000 S4 PE 150 platform in Centre d'expertise et de services Génome Québec. Our in‐house pool‐seq pipeline (Lind 2021a, 2021b) was employed to align the reads to the reference genome and call SNPs. For raw SNPs, only bi‐allelic loci in regions without annotated repetitive elements or potentially paralogous genes were retained. The annotated repetitive elements were acquired from the LP genome annotation (Wegrzyn et al. 2014). The potentially paralogous genes were identified as described in Lind et al. (2022) using haploid megagametophyte sequences, for the heterozygous SNP calls for haploid sequences are likely to represent misalignments of paralogs. Afterwards, the SNP loci with depth (DP) < 10, DP > 400, global minor allele frequency < 0.05, or > 25% missing data were also removed.

### 2.3 Genetic Structure Analyses

The genetic structure among the 160 pooled samples, which represented four pooled libraries (D1‐R, D1‐S, D2‐R, D2‐S) for each of the 40 seedlots, was detected by using principal component analysis (PCA), reconstructing a phylogenetic tree, and by assessing correlation of allele frequencies and population differentiation (*F*_ST_) between samples using unlinked SNPs. To reduce the impact of linkage disequilibrium on estimation, the SNP set was thinned using vcftools (Danecek et al. 2011), so that no two sites were within 100,000 bp. PCA and genetic distance were calculated using the major allele depth and the R package “adegenet” (Jombart 2008). An unrooted phylogenetic tree was constructed using the distance output and the Neighbour‐Joining algorithm, which was implemented by the R package “ape” (Paradis and Schliep 2019). A bootstrap analysis was performed using 5000 bootstrap replicates. Correlation coefficients of major allele frequencies between all pairs within the 160 samples were calculated and plotted using the R package “corrplot” (Wei and Simko 2017). *F*_ST_ was estimated between all pairs within the 160 samples using the R package “poolfstat” (Hivert et al. 2018). The clustering patterns output from these genetic structure analyses can be used to judge the subtle population structure leading to false positive problems in GWAS.

### 2.4 Dissection of Genetic Architecture Underlying *D. septosporum* Response

The alleles and candidate genes associated with *D. septosporum* response were detected using the pool‐GWAS method. A GWAS case–control approach was developed using pool‐seq samples to identify allele frequency differences between susceptible and resistant pines inoculated with *D. septosporum*. This method was based on the Cochran–Mantel–Haenszel (CMH) method and implemented in our in‐house pipeline (Lind 2021a, 2021b). While sequencing read depth was commonly used as an estimation of allele count in such applications of the test (Futschik and Schlötterer 2010; Schlötterer et al. 2014), reliable results would be only achieved when the haploid size of the sample is much greater than the depth of coverage (so that most reads arise from uniquely sampled haplotypes). As the pool size used in the present study (diploid size of 2*N* = 10) is often smaller than the sequencing depth of coverage, read counts were converted into allele frequencies and then multiplied by the ploidy to yield an approximate count for each pool that represents the real replication level. A simulation was conducted to compare the false positive rate from the CMH test using uncorrected or corrected allele counts across a range of numbers of demes, individuals, and depths. Results showed that the false positive rate of the CMH method is high, especially when the sequencing depth is much higher than the pool size (Figure S2a). Using corrected allele counts, the allele frequencies multiplied by the ploidy (*N* = 10) tend to lower the false positive rate (Figure S2b). Thus, the corrected allele counts were used for the CMH test.

As the genetic structure analysis showed that LP × JP and JP samples tend to cluster, JP samples and LP × JP samples were combined (JP + LP × JP) for downstream analyses. Pool‐GWAS was performed separately within LP and JP + LP × JP samples. Analysis of R and S samples was conducted by combining individuals inoculated with D1 or D2 isolates to maximize statistical power. To identify candidate genes, SNPs in the top 1% of −log_10_(*p*) values from a pool‐GWAS test were classified as outliers. As linkage disequilibrium can amplify signatures of selection, candidate resistance genes were identified as those with a large number of outlier SNPs relative to the average genome‐wide expectation, as represented by an index based on the binomial distribution as per Yeaman et al. (2016). The identified loci on the enriched genes were realigned to the 12 LP linkage groups (MacLachlan et al. 2021), and Manhattan plots were drawn. The LP transcriptome (Lu et al. 2021) was used to annotate the reference genome. The aligned transcripts to the reference genome were identified as those with a minimum alignment identity of 90% and a minimum alignment coverage of 85% using GMAP v2019‐03‐15.

### 2.5 Linkage Disequilibrium (LD) Between Loci

A non‐linear model (Hill and Weir 1988) was used to estimate the decay of LD with physical distance. Pairwise correlation (*r* ^*2*^) of allele frequencies between loci on the same scaffold was calculated. The *r* ^*2*^ values and the physical distances between loci on all scaffolds (genome‐wide) or on scaffolds containing the identified top candidate genes (significant) were used to fit the non‐linear model as described by Marroni et al. (2011). The LD decay graph was plotted using R (R Core Team 2018).

### 2.6 Introgression

To study patterns of introgression affecting different regions of the genome and among different samples, the mean pairwise *F*_ST_ was calculated for each locus using the R package “poolfstat.” Loci with high *F*_ST_ values have high genetic differentiation, which may represent species barriers or genomic regions under divergent selection, while loci with low *F*_ST_ values imply introgression. To estimate genetic similarity of the studied JP and LP seedlots, the five JP samples (p36 to p40) and six pure LP samples (the six westmost LP samples, p15, p16, p18, p20, p23, p25) were used to calculate *F*_ST_ values, which were then averaged. To evaluate whether regions associated with *D. septosporum* response have atypical patterns of introgression, the *F*_ST_ values of the top 1% *D. septosporum* response outliers identified from LP (*N* = 3358) and those from JP + LP × JP (*N* = 3041) were compared with *F*_ST_ values of randomly drawn SNPs (*N* = 3400).

Similarly, *F*_ST_ between the JP + LP × JP samples (p29 to p40) and six pure LP samples (p15, p16, p18, p20, p23, p25) was averaged to estimate genetic similarity of the studied JP + LP × JP and LP. Different genomic regions were represented by different SNP sets, including *D. septosporum* response outliers identified from LP (*N* = 3358) and outliers identified from JP + LP × JP (*N* = 3041), as well as the unlinked SNP set (*N* = 31,716), which was used in 2.3 for detecting genetic structure and representing random genomic regions. The genetic similarity (*F*_ST_) of JP + LP × JP and LP was regressed to the longitude of these JP + LP × JP samples. The regression slopes for the three SNP sets were compared using the analysis of covariance method implemented by the R package “lsmeans” (Lenth 2016; R Core Team 2018).

## 3 Results

### 3.1 Patterns of Genetic Structure

**FIGURE 2:**
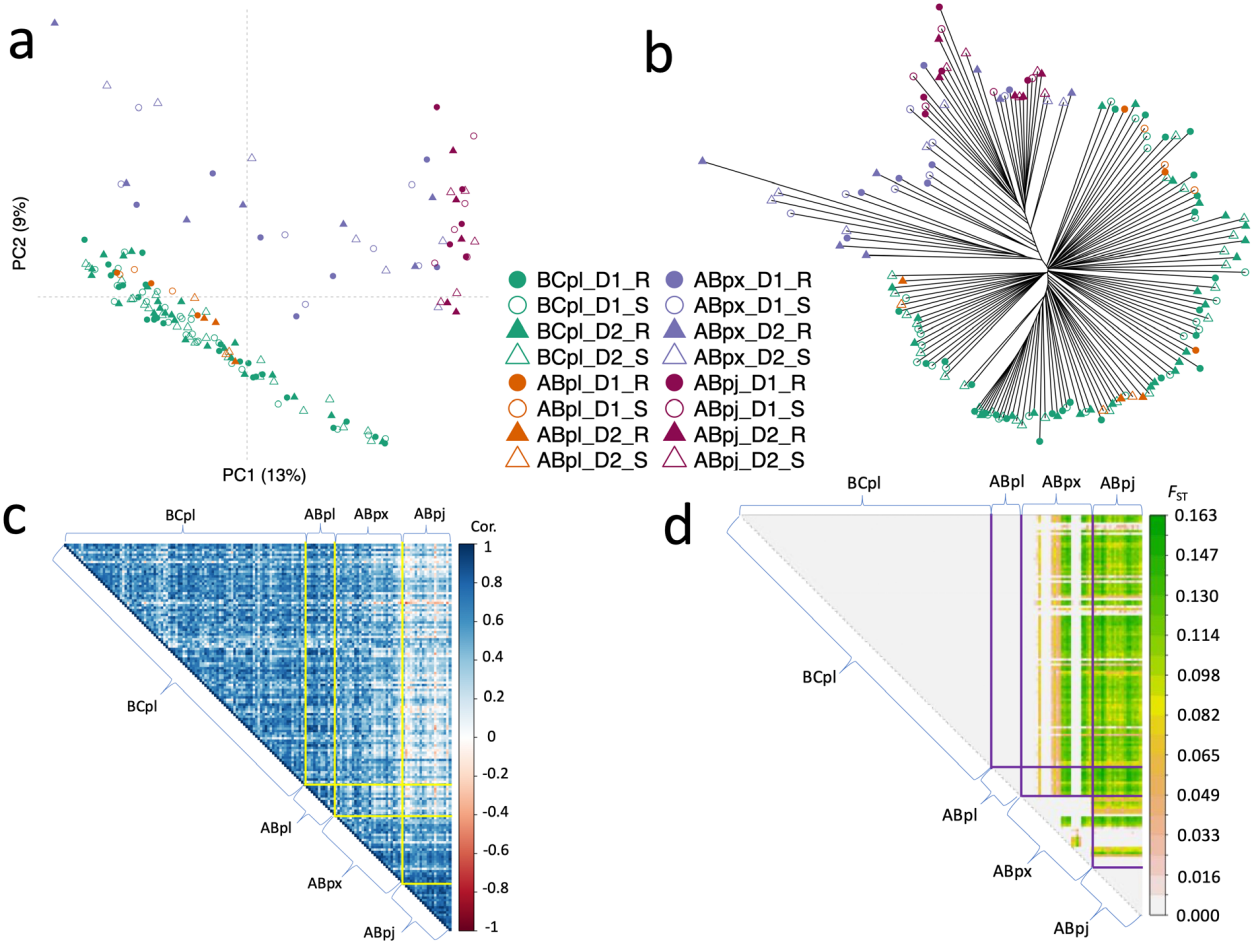
Genetic structure among the 160 pooled samples. (a) Principal component analysis. (b) Unrooted phylogenetic tree. The bootstrap values for each branch were shown in Figure S4. (c) Correlation plot of the major allele frequencies between samples. (d) Pairwise *F*_ST_ values between pools. BC_LP— *Pinus contorta* (lodgepole pine) samples from British Columbia; AB_LP— *Pinus contorta* (lodgepole pine) samples from Alberta; AB_LPxJP— *Pinus contorta* × *P. banksiana* hybrid samples from Alberta; AB_JP— *Pinus banksiana* (jack pine) samples from Alberta. D1—*D. septosporum* isolate 1; D2—*D. septosporum* isolate 2. R—resistant tree; S—susceptible tree. In (c) and (d), each column represents a pooled sample. The samples were arranged from left to right according to the longitude of the sampling location, and four pooled libraries per seedlot (D1‐R, D1‐S, D2‐R, D2‐S) were arranged together.

After filtering, a total of 364,691 SNP loci were retained for downstream analyses, and most loci had a minor allele frequency between 0.05 and 0.1 (Figure S3). A thinned set of 31,716 unlinked SNPs was used for studying genetic structure among the 160 pooled samples (Figure 2). The most prominent patterns of structure were associated with species (Figure 2a,b, Figure S4), with LP samples readily distinguishable from JP and LP × JP samples. LP × JP samples clustered more closely with JP than LP. Compared to those sampled from the eastern hybrid zone, western LP × JP tend to be more similar to LP (samples were arranged according to longitude from left to right in Figure 2c,d). Given this clear pattern of genetic structure, we conducted GWAS in LP and JP + LP × JP samples separately.

### 3.2 Signatures of Association to Infection Response

**FIGURE 3:**
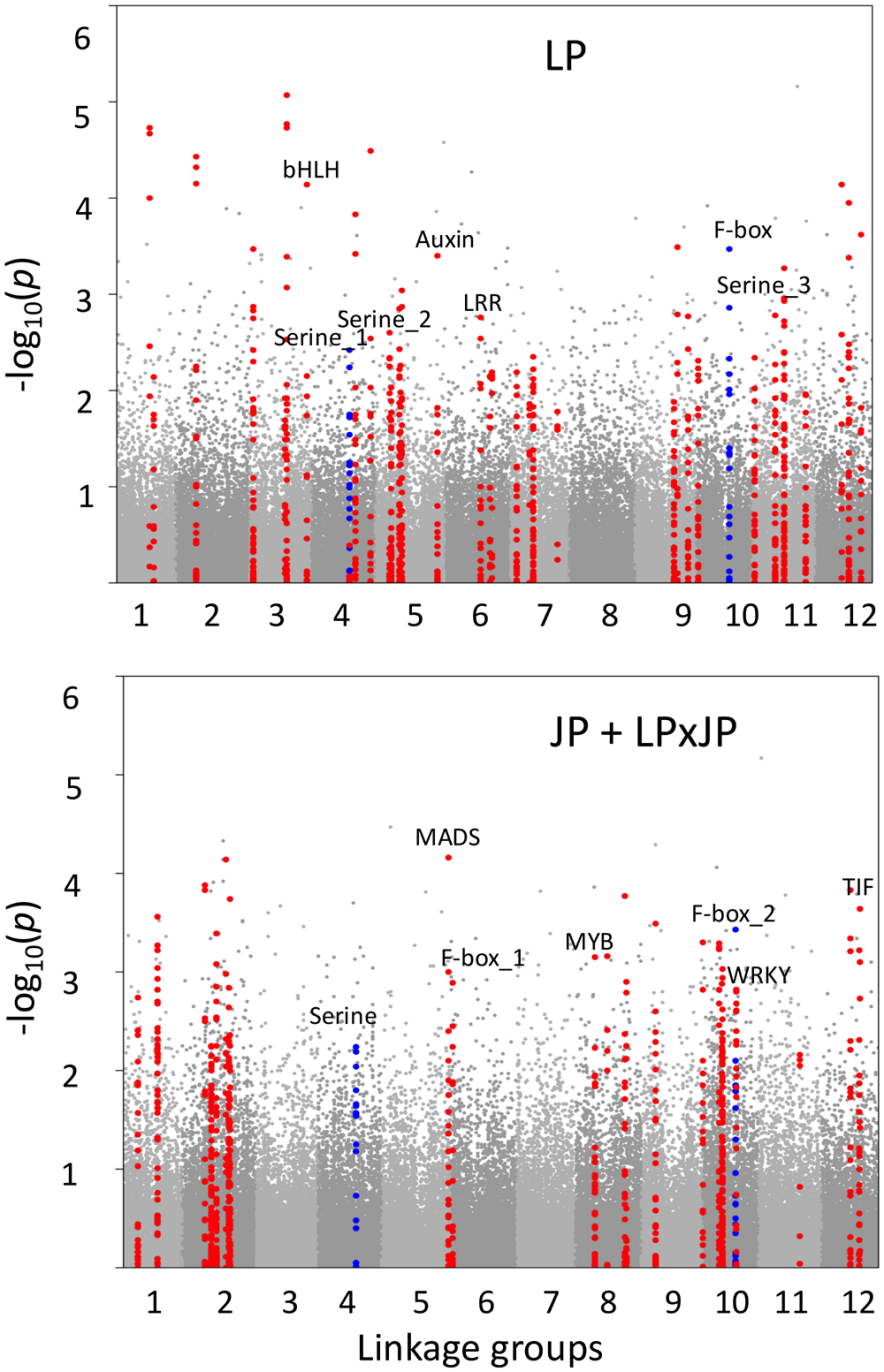
Manhattan plot showing SNPs associated with *D. septosporum* response within *Pinus contorta* (lodgepole pine, LP) and combined *Pinus banksiana* and *Pinus contorta* × *P. banksiana* hybrids (JP + LPxJP) samples. The *x* axis represents 12 linkage groups. The *y* axis represents significance of associations identified from pool‐GWAS. Candidate genes were identified using a binomial test for enrichment of outlier SNPs per gene. Candidate genes and their surrounding 50 kbp regions are highlighted in red. Of the three top‐ranked candidate genes identified within both the LP and JP + LPxJP samples, one was not present in the linkage map, while the other two are highlighted in blue (“Serine” on linkage group 4 and “F‐box” on linkage group 10). Labels in the graphs represent candidate genes encoding plant‐pathogen interaction proteins: bHLH—bHLH transcription factor; Serine—serine/threonine‐protein kinase; Auxin—auxin response factor; LRR—proteins with leucine‐rich repeat; F‐box—F‐box Kelch‐repeat protein; MADS—MADS transcription factor; MYB—MYB transcription factor; WRKY—WRKY transcription factor; TIF—translation initiation factor. The candidate gene IDs (in Table S2) are: In LP, bHLH—Scaffold_2015‐465162‐569336; Serine_1—Scaffold_3728‐494595‐736720; Serine_2—Scaffold_3141‐1‐105566; Serine_3—Scaffold_1072‐1299306‐1374553; Auxin—Scaffold_3823‐1571412‐1689102; LRR—Scaffold_1361‐487806‐570406; F‐box—Scaffold_3698‐733755‐839782; In JP + LPxJP, Serine—Scaffold_3728‐494595‐736720; MADS—Scaffold_427‐2324701‐2717580; F‐box_1—Scaffold_3698‐733755‐839782; MYB—Scaffold_4478‐441537‐627389; F‐box_2—Scaffold_342‐1965787‐2253515; WRKY—Scaffold_830‐1136302‐1239031; TIF—Scaffold_207‐7583953‐7695067.

Out of the 100 top‐ranked candidate genes in each test (Table S2), three were identified within both the LP and JP + LP × JP samples (Figure 3). This is significantly more than the hypergeometric expectation, based on the probability of overlap for two draws of 100 genes from 20,026 genes. A previous study anchored 10,093 scaffolds on the LP linkage map (MacLachlan et al. 2021), so we plotted SNPs and labeled the enriched plant‐pathogen interaction candidate genes on the 12 linkage groups (Figure 3). The three genes identified in both species encode ABC transporter, F‐box Kelch‐repeat protein, and serine/threonine protein kinase. Genes encoding F‐box protein and serine protein kinase were highlighted in Figure 3. Genes encoding ABC transporter were not included in the linkage groups, so they were not included in Figure 3. We also checked the enrichment of differentially expressed genes (DEGs) in the top 100 ranked genes. Out of 631 DEGs identified in the previous study (Lu et al. 2021), four were also candidate genes in the present study. Fisher's Exact test showed there is no significant enrichment of DEGs in the candidate genes.

**FIGURE 4:**
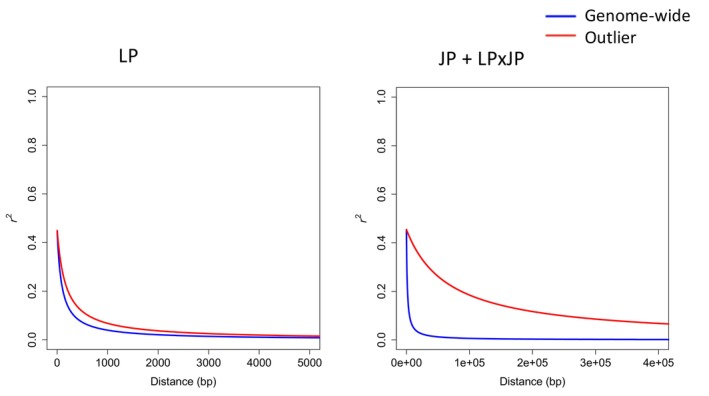
Linkage disequilibrium (LD) decay for genome‐wide loci and outlier loci identified from *Pinus contorta* (lodgepole pine, LP) and combined *Pinus banksiana* and *Pinus contorta* × *P. banksiana* hybrids (JP + LPxJP) samples. The *r* ^*2*^ values (pairwise correlation of allele frequencies between loci) and the physical distances between loci were used to fit the non‐linear model as described by Marroni et al. (2011).

Loci on scaffolds with outliers (the SNPs with top 1% of −log_10_(*p*) values from pool‐GWAS test) tend to exhibit a more gradual decay in LD compared with the genome‐wide loci on all scaffolds (Figure 4), with JP + LP × JP pine outliers having a much slower decay rate than LP. In LP, the distance at which LD decays to half of its maximum value is 96 bp for genome‐wide loci, and 174 bp for outlier loci. In JP + LP × JP, the half LD decay distance is 1357 bp for genome‐wide loci, and 68,215 bp for outlier loci. The slower LD decay pattern for the outliers may indicate selection on these identified loci.

### 3.3 Introgression

**FIGURE 5:**
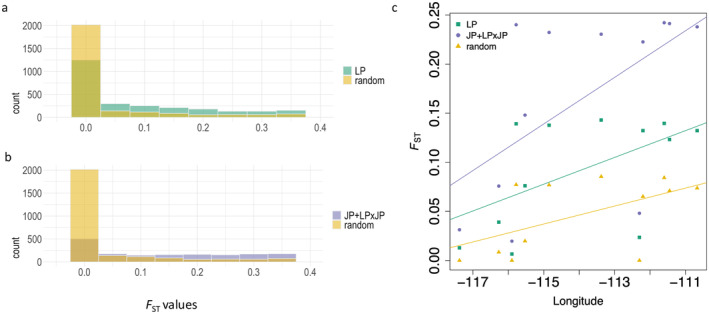
*F*_ST_ values of *D. septosporum* response outliers (a & b) and regression patterns of *F*_ST_ values with longitude. For a & b, the *F*_ST_ values were calculated between the *Pinus banksiana* (jack pine, JP) samples (p36 to p40) and the six pure *Pinus contorta* (lodgepole pine, LP) samples (p15, p16, p18, p20, p23, p25). JP + LPxJP represents the combined JP and *Pinus contorta* × *P. banksiana* hybrid samples. *F*_ST_ values of *D. septosporum* response outliers identified from LP (*N* = 3358) and JP + LPxJP (*N* = 3041) samples were compared with *F*_ST_ values of randomly drawn SNPs (*N* = 3400). Outliers identified from both species have higher *F*_ST_ values than randomly drawn SNPs (*p*‐value < 0.01). For c, the *F*_ST_ values were calculated between the JP + LPxJP samples (p29 to p40) and the six pure LP samples (p15, p16, p18, p20, p23, p25) and then averaged for each JP + LPxJP sample. Different genomic regions were represented by different SNP sets, including LP outliers (*N* = 3358), JP + LPxJP outliers (*N* = 3041), and unlinked SNPs (*N* = 31,716). The *F*_ST_ values represented by different SNP sets were regressed to the longitude of these JP + LPxJP samples. Differences between slopes are not significant (ANCOVA, *p*‐value > 0.3).

By calculating average *F*_ST_ between pairs of LP and JP samples, we found that the *D. septosporum* response outliers, which were identified separately in LP or JP + LP × JP samples, have higher *F*_ST_ values than randomly drawn SNPs (Wilcoxon test *p*‐value < 0.01, Figure 5a,b), with outliers identified in JP + LP × JP samples showing higher *F*_ST_ values than those identified in LP samples (Wilcoxon test *p*‐value < 0.01). These results suggest that the outliers associated with disease response constitute or are linked to genomic regions for divergent selection or species barriers, rather than facilitating introgression. *F*_ST_ estimates between JP + LP × JP samples and a set of pure LP samples showed a pattern of increased differentiation with distance eastwards of the JP + LP × JP samples (Figure 5c), which were higher for the outliers than randomly chosen SNPs. Given the small number of samples, significance of linear model fitting is borderline, with *p*‐value = 0.06 for outliers identified in LP, *p*‐value = 0.05 for outliers identified in JP + LP × JP, and *p*‐value = 0.06 for randomly chosen SNPs (df = 10). The differences between regression slopes on different gene sets (LP outliers, JP + LP × JP outliers, and unlinked SNPs) were not significant (*p*‐values of pairwise comparisons > 0.3). Though it is not possible to test whether a linear vs. discontinuous model would fit better to these data, the results suggest that isolation‐by‐distance is occurring here.

## 4 Discussion

In recent decades, the fungal pathogen *D. septosporum* has become an increasing threat to LP in British Columbia (Feau et al. 2021; Welsh, Lewis, and Woods 2009). Although *Dothistroma* needle blight (DNB) has not been observed in natural stands of JP in Alberta, Ramsfield, Myrholm, and Tomm (2021) confirmed JP's susceptibility to infection by *D. septosporum* using a trial under natural conditions. The potential of climate change to allow this pathogen to spread across the wide natural distribution of JP raises serious concerns. The hybrid zone of contact between the sister species LP and JP may act as a bridge, allowing this fungal pathogen host shift (Taylor, Larson, and Harrison 2015), as evidenced by the intermediate phenotype of gall development in LP × JP seedlings compared to that in LP and JP (McAllister et al. 2022). The genetic basis of pathogen resistance in each species is therefore an important factor affecting the potential for host shift. To study the genetics of resistance to DNB, we analyzed the genetic architecture underlying *D. septosporum* response using the studied samples of LP, JP, and LP × JP. We identified candidate genes associated with *D. septosporum* response and inferred the impact of introgression on the genetic structure underlying *D. septosporum* response in the studied samples. We detected largely non‐overlapping sets of candidate genes in LP and JP + LP × JP in response to *D. septosporum*, which might be shaped by different evolutionary forces of selection and introgression. Alternatively, the non‐overlapping sets of candidate genes might reflect that many non‐causal genes exist in the top 1% of GWAS outliers of each species, given that we did not test for significance at the gene level. In either case, it seems clear that resistance to DNB is a highly polygenic trait with alleles of small effect.

This study is the first report of genome‐wide scanning of *D. septosporum* response genes in LP, JP, and LP × JP. We identified a few top candidate genes encoding leucine‐rich repeats (LRR)‐containing protein domains and serine‐threonine kinases in LP samples (Figure 3, Table S2). Plant breeders have long used nucleotide‐binding LRR (NB‐LRR) receptor R genes for preventing or reducing diseases in crops. When attacked by infectious pathogens, plant NB‐LRR receptors can recognize pathogen effectors by either direct or indirect mechanisms (Dodds and Rathjen 2010). Such a strategy is called effector‐triggered immunity (ETI), which occurs after a basal resistance, pathogen‐associated‐molecular‐patterns‐triggered immunity (PTI). In a previous study using RNA‐seq data, Lu et al. (2021) identified genes involved in PTI and ETI, as well as R genes with positive selection signals in LP. These results indicate that the long coevolutionary history between LP and *D. septosporum* (Capron et al. 2021; Welsh, Lewis, and Woods 2009) has given rise to some tolerance or resistance mechanism against DNB. Since most of the identified associated markers are likely not causal but rather are in LD with the causal loci, the observed fast LD decay rate in lodgepole pine implies promising fine mapping resolutions, as the identified markers are likely to be proximal to the causative genes.

On the contrary, JP, as a potential new host, may not have coevolved such a specific defense, and the long LD blocks surrounding candidate genes identified from JP + LP × JP samples, which are typical of hybrid zones, limit the resolution of association mapping, so it may be difficult to locate the causative genes for follow‐up studies (Goulet, Roda, and Hopkins 2017; Wilson and Goldstein 2000). Additionally, we caution that the capture probes were designed using LP not JP genes, ascertainment bias might give rise to the underrepresentation of JP alleles (Lachance and Tishkoff 2013). However, we can use these JP + LP × JP candidate genes to infer how introgression affects the frequencies of disease‐resistant alleles. Since we analyzed pool‐seq data and lacked the pure JP samples out of the hybrid zone, it is not possible to analyze parental origin information, thus we inferred the ancestry using clustering. The clustering patterns in Figure 2 show that most hybrid pine trees in our study had greater JP than LP ancestry, likely in part due to our limited sampling of JP from further Eastwards. Western LP × JP tend to be more genetically similar to LP, while eastern LP × JP tend to be more genetically dissimilar to LP. This is a typical characteristic for ongoing introgression and hybridization in the contact zone with a possible trend of LP expanding its range eastward (Harrison and Larson 2014; Moran et al. 2021).

In hybrid zones, alleles in some genomic regions are able to introgress across species boundaries, whereas alleles that constitute species barriers or are under divergent selection will remain differentiated (Harrison and Larson 2014). We found significantly high *F*_ST_ values between JP and LP samples for the candidate genes associated with *D. septosporum* infection response. This can occur if these candidate genes tend to reside in genomic regions with restricted gene flow, driven by linkage with genes for divergent selection or species barriers. McAllister et al. (2022) observed a gradient of resistance to *C. harknessii* in LP, which could be associated with introgression of resistance genes from JP into LP. Since LP might have a long coevolutionary history with *D. septosporum* (Capron et al. 2021; Welsh, Lewis, and Woods 2009), the introgression of LP genes into hybrids may increase resistance to *D. septosporum*. However, variation in *F*_ST_ along longitude (Figure 5c) did not differ significantly between background and candidate loci, so we cannot draw strong conclusions about adaptive introgression here.

## 5 Conclusion

The recent outbreaks of DNB in LP and its ability to infect JP pose a pressing need to understand the genetic architecture of resistance to *D. septosporum*. The present study provides an array of candidate genes associated with *D. septosporum* response using pool‐GWAS. The identified candidate genes within LP and JP + LP × JP samples are largely non‐overlapping, and the adaptive introgression is yet to be found. This knowledge can be used to develop genomic tools to minimize DNB risk and guide forest management strategies. Further testing and validation of these associations using a SNP array is currently underway.

## Conflicts of Interest

The authors declare no conflicts of interest.

## Supporting information


Figure S1.

Table S1.

## Data Availability

The raw pool‐seq data were deposited in NCBI SRA (accession number: PRJNA602898; http://www.ncbi.nlm.nih.gov/sra). The assembled transcriptomes and their annotation were deposited in Dryad https://doi.org/10.5061/dryad.prr4xgxtk. The scripts used for analyses were deposited in https://github.com/Mengmeng‐Lu/Genetic‐architecture‐underlying‐response‐to‐the‐fungal‐pathogen‐Dothistroma‐septosporum‐in‐Pinus‐con.
